# Defining Microbiome Health through a Host Lens

**DOI:** 10.1128/mSystems.00155-19

**Published:** 2019-05-14

**Authors:** Sean M. Gibbons

**Affiliations:** a Institute for Systems Biology, Seattle, Washington, USA; b eScience Institute, University of Washington, Seattle, Washington, USA

**Keywords:** ecology, evolution, health, host response, microbiome

## Abstract

We are walking ecosystems, inoculated at birth with a unique set of microbes that are integral to the functioning of our bodies. The physiology of our commensal microbiota is intertwined with our metabolism, immune function, and mental state. The specifics of this entanglement remain largely unknown and are somewhat unique to individuals, and when any one piece of this complex system breaks, our health can suffer. There appear to be many ways to build a healthy, functional microbiome and several distinct ways in which it can break. Despite the hundreds of associations with human disease, there are only a handful of cases where the exact contribution of the microbiome to the etiology of disease is known. Our laboratory takes a systems approach, integrating dynamic high-throughput host phenotyping with eco-evolutionary dynamics and metabolism of gut microbiota to better define health and disease for each individual at the ecosystem level.

## Abstract (precis)

We are walking ecosystems, inoculated at birth with a unique set of microbes that are integral to the functioning of our bodies. The physiology of our commensal microbiota is intertwined with our metabolism, immune function, and mental state.

## PERSPECTIVE

The one-pathogen–one-disease paradigm—a major focus of medical microbiology for more than a century—has been complicated by the discovery of the human microbiome (1). The gut microbiome is a crucial subsystem of the body, intimately tied to the development of our immune system, our physiology, and even our psychology (1). A breakdown in the ecological structure of our gut has been associated with inflammatory disorders, metabolic syndromes, and cancer (2). Ecological restoration of the gut through fecal microbiota transplantation (FMT) from a healthy donor has proven to be an effective, population-scale treatment for Clostridium difficile infections (3). However, we do not fully understand the mechanisms underlying FMT efficacy, which makes it difficult to translate such treatments to other disease types. In particular, microbial therapeutics that work in one individual are often ineffective in another, suggesting that a personalized approach is necessary for many microbiome health issues. Our interdisciplinary research group at the Institute for Systems Biology (ISB) focuses on integrating dense, longitudinal host phenotype data (i.e., genetics, blood proteomics and metabolomics, clinical labs, diet and lifestyle questionnaires, and physical activity measurements) with eco-evolutionary dynamics of gut microbiota to help build dynamic, multispecies, ecosystem-level models for individual humans. We have partnered with the personalized wellness company Arivale (Seattle, WA), which streamlines participant recruitment, sample acquisition, and data generation. More than 90% of Arivale customers allow their data to be used for research purposes—currently >5,000 people. The longitudinal and multiomic nature of these data is crucial for establishing putative directionality of associations between host and microbial factors in high throughput. Ultimately, we will use these large directed networks to inform experiments and trials and to develop personalized ecological therapeutics to treat complex conditions. Recently, ISB has partnered with the Providence health care system (>50 hospitals in the western United States) to translate our emerging science into the clinic. While the promise of personalized ecological therapeutics is great for a range of conditions, we are still far from being able to rationally engineer the ecology of the gut to improve human health. In order to build a toolset for engineering the gut microbiome we must first understand the basic processes underlying community assembly, stability, and function.

## WHAT ARE THE RULES?

Identification of the determinants of gut community assembly, stability, and function across the life span of a host is an important scientific and medical challenge. Each of us contains a unique set of bacterial strains obtained from our mothers (4) or picked up from the environment (5, 6), which can remain with us for years (7). Persistent strains evolve and adapt to us over the course of our lives (7, 8), with unknown consequences. The ability of a commensal to engraft in a given individual is controlled by a variety of factors, including the immune system (9), availability of metabolic niche space (10), and competition with indigenous microbiota (7, 10). Bacterial species in the gut maintain similar abundance levels across humans (11), suggesting that species-specific niche volumes are conserved within the host population. Despite apparent similarities in the size of a given commensal niche across people, each person harbors a unique complement of species, which is stable over months to years despite frequent shifts in diet and host health (11). Combined, these observations suggest that many commensal species are functionally redundant and that each host harbors a unique set of niches. Thus, the gut microbiome is a self-assembled “organ” specifically tailored to each of us. Our lab is focused on understanding how individual-specific ecological and evolutionary forces contribute to the stability and functionality of the gut microbiota and how differences in the structure and composition of the microbiome contribute to human health and disease.

## WHAT IS A HEALTHY MICROBIOME?

There seem to be many ways of constructing a “healthy” microbiome. While everyone is a unique microbiome snowflake at the species or strain level, healthy people harbor a minimal set of microbiota that saturate functional niches necessary to maintain wellness. Thus, it makes sense to define ecosystem health at the functional, rather than taxonomic, level. There are a few dominant modes for how a healthy microbiome can break down (2). First, crucial functional capacity can be lost from the system, either as a cause or consequence of disease, as in the case of depleted butyrate-producing taxa in inflammatory bowel disease (2). Second, pathogenic taxa can invade and engraft in the gut, as in the case of C. difficile infections (2). Invasion of pathogens is often associated with severely depleted species diversity in the gut (2, 3), which suggests that opportunistic pathogens are able to invade empty niches when alpha-diversity drops below some critical threshold. Our research group looks at microbiome health through the lens of quantitative host phenotyping. Specifically, we have found strong associations between circulating blood metabolites and the structure of the gut microbiome. In particular, we can predict gut microbiome alpha-diversity using a subset of 11 blood metabolites, independent of the exact species composition of an individual’s microbiota (12). Thus, we suggest that certain aspects of microbiome “health” can be inferred from the blood metabolome, where loss of critical functional capacity in the gut can be sensed through its impact on host physiology. We develop methods for identifying critical metabolic and immunomodulatory associations between humans and their microbiota from rich multiomic time series data. We are designing tools for inferring directedness of associations between host and microbiome features, taking advantage of the order of events in time. We will test these putatively causal, directed relationships in human feeding studies and clinical trials. By taking a personalized, systems biology approach to host-microbe symbiosis, we hope to expand and strengthen the actionable links between gut microbiota and human health.

## HOW DO WE FIX A BROKEN SYSTEM?

The major challenges, as outlined above, are to understand how to manipulate the ecology of the gut within and across individuals and to define what it means for the microbiome to “break.” While we are still in the early phase of addressing these challenges, there are a few clear paths toward developing effective ecological therapeutics that we plan to explore.

First, there is a clear association between low gut microbiome diversity and susceptibility to pathogen invasion and infectious disease. In the case of C. difficile, we have shown that patients with severely depleted species diversity in the gut are unlikely to recover to a healthy state following vancomycin treatment and are predisposed to recurrent infections (13). Recurrent C. difficile infections, which can now be resolved by FMT, place patients at significant risk of morbidity/mortality (13). We are developing clinical measures of gut diversity (12) for stratifying low- and high-diversity patients to prevent recurrent C. difficile cases altogether by promoting FMTs as a first-line therapy in low-diversity patients. Other approaches could be taken to prevent diversity collapse in the first place. For example, autologous fecal transplants could be routinely employed following antibiotic treatment to repopulate the gut with commensal bacterial species that might otherwise have been lost. The maintenance of diversity and niche saturation in the gut will play an important role in disease prevention.

Second, functional niche saturation of an individual’s microbiome appears to be related to health. Vacant niches could be identified in the microbiome itself or detected indirectly through metabolic or immunological perturbations in the host (12). If a crucial metabolic niche important for host health is unoccupied, targeted probiotic interventions could be deployed to recover these missing microbes. Prior work has shown that probiotics will engraft in the human gut if their metabolic niche is vacant (10). Certain microbial niches important to host phenotypes will be conserved at the population level, while others may vary from person to person. Obtaining a healthy reference point for an individual, both in terms of their microbiome and their physiological state, may be important for monitoring person-specific deficits that might arise later in life. One interesting challenge to personalize probiotic cocktails is the fact that our indigenous taxa have evolved to each of us over many years (7) and may be difficult to displace with host-naive strains. FMT data indicate that short-term host-naive strain engraftment is common (3), but indigenous strains may outcompete these naive strains over longer timescales. Personalized adaptation of the microbiota might be important for maintaining ecological stability and health (7). Thus, it may be prudent to periodically biobank our stool when we are healthy to conserve this personalized ecological and evolutionary diversity.

Third, a promising approach to engineering the microbiome is the introduction of an exclusive metabolic niche (14, 15). As stated above, engraftment of a bacterium into an intact microbiota requires that its niche is both present and available (10). It is possible to introduce novel niches into the gut that are inaccessible to the indigenous microbiota (i.e., “orthogonal niches”). These orthogonal niches allow for the reversible engraftment of synthetic gut microbes. As a proof of concept, we achieved stable and reversible engraftment in a mouse model through the coadministration of seaweed and a human Bacteroides plebeius strain with the metabolic capacity to degrade porphyrin molecules in seaweed (14). This method for manipulating the microbiome does not rely upon understanding endogenous niche structure, and the introduced organisms can be genetically engineered to carry out specific therapeutic functions. However, caution is necessary, as little is known about how the host immune system or the indigenous microbiota might react to the long-term engraftment of a synthetic taxon.

## CONCLUSION

Before we can integrate ecological therapeutics into modern medicine, we must uncover the basic rules governing the ecology and evolution of gut commensals within and across hosts. We must draw a detailed map for how variation in the microbiome is associated with variation in host physiology, immunity, and overall wellness. To further this goal, our lab develops computational and wet-lab approaches for integrating microbial community ecology, population genetics, and classical systems biology to build a system-level understanding of human health and disease.
